# 27. Co-infections and antimicrobial use in patients hospitalized with COVID-19

**DOI:** 10.1093/ofid/ofab466.027

**Published:** 2021-12-04

**Authors:** Prithiv Prasad, Ioannis Zacharioudakis, Jordan Poles, Yanina Dubrovskaya, Dinuli Delpachitra, Eduardo Iturrate, Sigridh Munoz-Gomez

**Affiliations:** 1 NYU Grossman School of Medicine , New York, NY; 2 New York University Grossman School of Medicine, New York, New York; 3 NYU Grossman School of Medicine, New York, New York; 4 NYU Langone Health, New York, New York; 5 NYU Long Island School of Medicine, New York, New York

## Abstract

**Background:**

In-hospital antimicrobial use among COVID-19 patients is widespread due to perceived bacterial and fungal co-infections. We aim to describe the incidence of these co-infections and antimicrobial use in patients hospitalized with COVID-19 to elucidate data for guiding effective antimicrobial use in this population.

**Methods:**

This retrospective study included all patients admitted with COVID-19 from January 1, 2020, to February 1, 2021 at any of the three teaching hospitals of the NYU Langone Health system. Variables of interest were extracted from the health system’s de-identified clinical database. The nadir of hospital admissions between the first and second peaks of hospital admissions in the dataset was used to delineate the First Wave and Late Pandemic periods of observation. A cut-off of 48 hours after admission was used to differentiate Co-infections and Secondary infections respectively among isolates of clinically relevant bacterial or fungal pathogens in blood or sputum samples. Population statistics are presented as median with interquartile range (IQR) or total numbers with percentages.

**Results:**

663 of 7,213 (9.2%) inpatients were found to have a positive bacterial or fungal culture of the respiratory tract or blood during the entire course of their initial admission at our hospitals for COVID-19. Positive respiratory cultures were found in 437 (6.1%) patients, with 94 (1.3%) being collected within 48 hours of admission. Blood culture positivity occurred in 333 patients (4.6%), with 115 (1.6%) identified within 48 hours of admission. Infection-free survival decreased with duration of hospitalization, with rate of secondary infections steadily rising after the second week of hospitalization as seen in Figure 1. 70.2% of inpatients received antimicrobials for a median duration of 6 antimicrobial days (IQR 3.0 – 12.0) per patient. A higher proportion of patients received antimicrobials in the first wave than in the late pandemic period (82.6% vs. 51.8%).

Table 1.

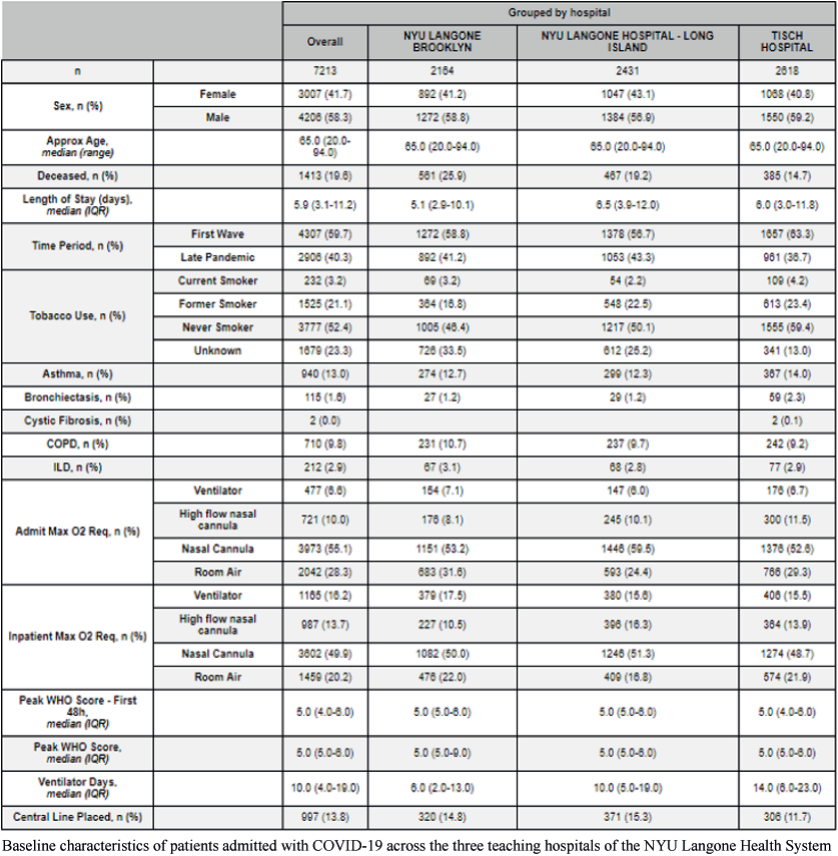

Table 2

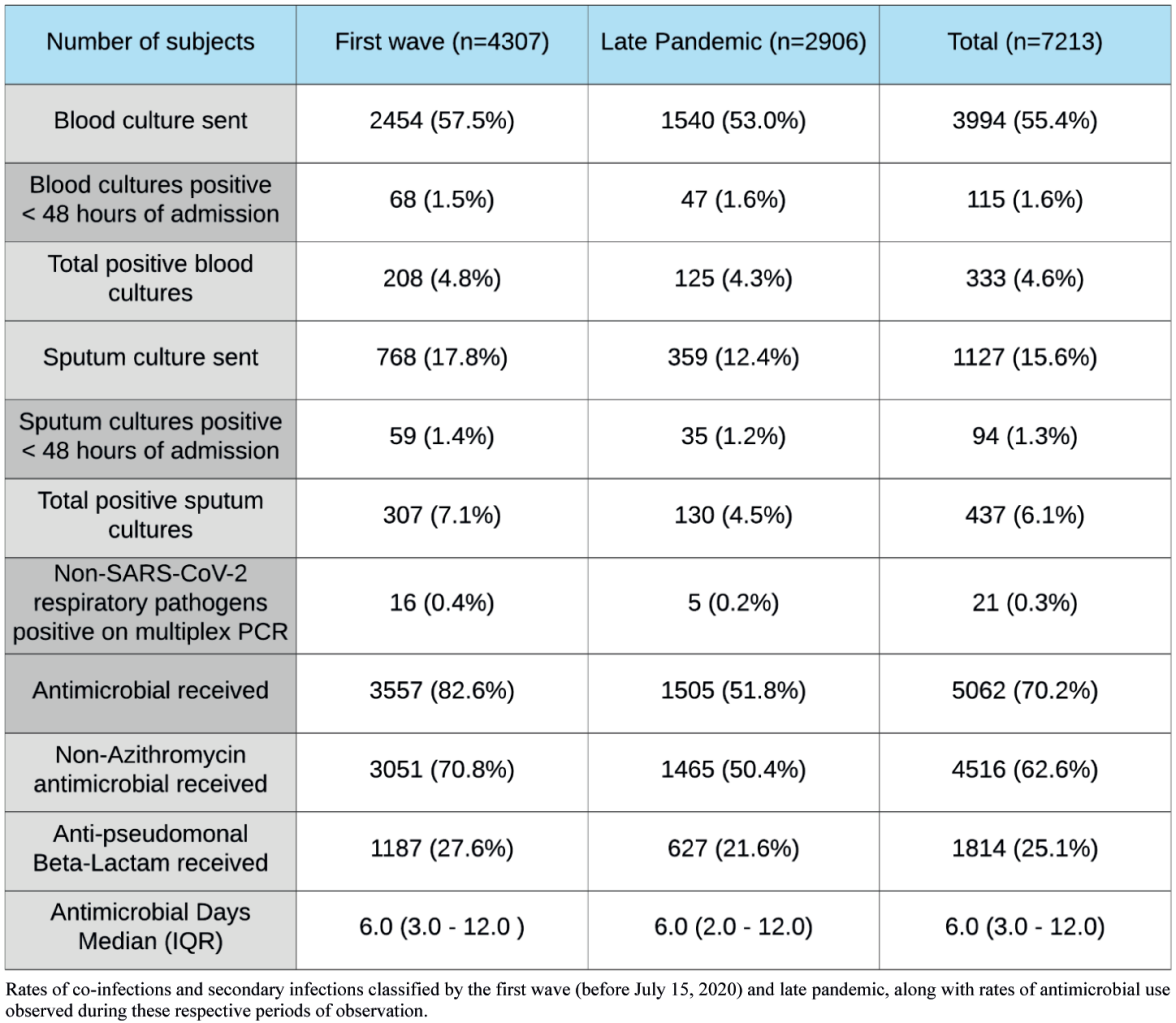

Figure 1

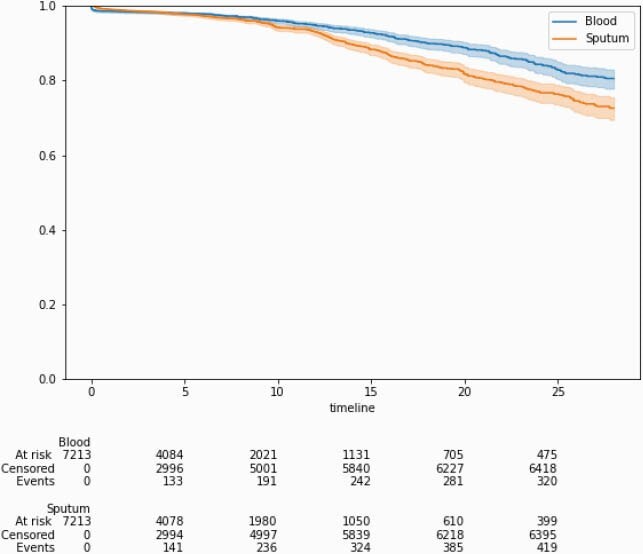

Infection free survival represented as duration of admission in days on the X-axis, and proportion of admitted patients remaining infection-free in the Y-axis. The blue line represents blood cultures and the orange line represents sputum cultures.

**Conclusion:**

There was a very low incidence of co-infection with SARS-CoV-2 infection at admission. A longer duration of hospitalization was associated with an increased risk of secondary infections. Antimicrobial use far exceeded the true incidence and detection of co-infections in these patients.

**Disclosures:**

**All Authors**: No reported disclosures

